# Diamine Oxidase from White Pea (*Lathyrus sativus*) Combined with Catalase Protects the Human Intestinal Caco-2 Cell Line from Histamine Damage

**DOI:** 10.1007/s12010-016-2390-3

**Published:** 2017-01-20

**Authors:** Catherine Jumarie, Marilyne Séïde, Lucia Marcocci, Paola Pietrangeli, Mircea Alexandru Mateescu

**Affiliations:** 1 Department of Biological Sciences and Centre TOXEN, Université du Québec à Montreal, CP 8888, Branch A, Montreal, Québec, H3C 3P8 Canada; 2 Department of Chemistry and Centre BioMed, Université du Québec à Montreal, CP 8888, Branch A, Montreal, Québec, H3C 3P8 Canada; 3 Department of Biochemical Sciences, “A. Rossi-Fanelli”, Sapienza University of Rome , P.le A. Moro 5, 00185 Rome, Italy; 4 Department of Chemistry, UQÀM, CP 8888, Branch A. Montreal, Québec, H3C 3P8 Canada

**Keywords:** Diamine oxidase, Catalase, H_2_O_2_, Histamine, Oxidative damage, Caco-2 cells

## Abstract

Diamine oxidase (DAO) administration has been proposed to treat certain gastrointestinal dysfunctions induced by histamine, an immunomodulator, signaling, and pro-inflammatory factor. However, H_2_O_2_ resulting from the oxidative deamination of histamine by DAO may be toxic. The purpose of this study was to investigate to which extent DAO from white pea (*Lathyrus sativus*), alone or in combination with catalase, may modulate histamine toxicity in the human intestinal Caco-2 cell line. The results show that histamine at concentrations higher than 1 mM is toxic to the Caco-2 cells, independently of the cell differentiation status, with a LC_50_ of ≅ 10 mM following a 24-h exposure. Depending on its concentration, DAO increased histamine toxicity to a greater extent in differentiated cells compared to undifferentiated cultures. In the presence of catalase, the DAO-induced increase in histamine toxicity was completely abolished in the undifferentiated cells and only partially decreased in differentiated cells, showing differences in the sensitivity of Caco-2 cells to the products resulting from histamine degradation by DAO (H_2_O_2_, NH_3_, or imidazole aldehyde). It appears that treatment of food histaminosis using a combination of vegetal DAO and catalase would protect against histamine toxicity and prevent H_2_O_2_-induced damage that may occur during histamine oxidative deamination.

## Introduction

Food histaminosis, associated with pseudo allergic phenomena, may occur upon ingestion of histamine-containing foods, such as red wine, beer, chocolate, sauerkraut, fish, and processed meats. Histaminosis is an enteric dysfunction that often affects the population in industrialized countries. This disorder is more frequent in people with low level of intestinal histaminase [1, 2], a copper-containing diamine oxidase (DAO, EC 1.4.3.22) able to catalyze the oxidative decomposition of histamine and producing H_2_O_2_, NH_3_, and imidazole acetaldehyde. The sensitivity to histamine is associated to symptoms such as gastro-intestinal discomfort [3, 4], migraine [5], irritation of nasal mucosa, itching, and other forms of allergy [6]. In addition, histamine, also known as a pro-inflammatory agent, is a risk factor for subjects suffering from inflammatory bowel diseases (IBDs) and colon cancer [7]. Furthermore, lowered activity of histamine catabolism has been shown in colonic mucosa of patients with colonic adenomas [8].

No pharmaceutical treatment is currently available to control the level of intestinal histamine. In this context, considering that the intestinal DAO is the main enzyme metabolizing ingested histamine [9], DAO supplements orally administered have been suggested for the treatment of food histaminosis and other dysfunctions related to impaired histamine metabolism. A commercial form of pig kidney DAO is available under different brand names as a food supplement (but not as pharmaceutical agent) aimed to break down food histamine in the digestive tract [1, 10, 11]. An oral form of a vegetal DAO, formulated as monolithic tablets with a carboxymethyl starch (CM-starch) excipient, was proposed to target the lower intestinal tract and to decrease the inflammation (i.e., Crohn’s disease, ulcerative colitis) [12].

However, considering that not only histamine as a substrate but also the products of DAO catalytic activity, especially H_2_O_2_, may be toxic to the cell, the aim of the study was to analyze the effect of histamine and of vegetal DAO activity on the viability of the human intestinal cells Caco-2. The effects of histamine-related molecules, such as histidine (precursor of histamine) and imidazole, were also investigated. Vegetal DAO was chosen for its much higher specific activity compared to that of animal DAO (i.e., pig kidney) [13–15] and also because enzymes from vegetal sources are more accepted by regulatory affairs. Histaminase was used alone or in combination with CAT. The rationale of using DAO in combination with catalase relies on the ability of CAT to decompose H_2_O_2_ into O_2_ and H_2_O. The investigation was conducted on undifferentiated (7-day-old) and differentiated (21-day-old) Caco-2 cell cultures. This cell line is widely used as an in vitro model to study intestinal function because it undergoes enterocytic differentiation spontaneously. An exponential growth phase is observed until dish culture confluence is reached (around day 7). Then, a stationary growth phase is observed and cells develop functional (i.e., transport mechanisms, enzymes) as well as morphological (microvilli) features of mature enterocytes [16, 17]. The level of differentiation increases up to 3 weeks of culture [18], and monolayers are homogenously polarized and differentiated by 4 weeks [19].

## Material and Methods

### Chemicals

Histamine, histidine, imidazole, hydrogen peroxide, o-phthaldialdehyde (OPT), 4-aminoantipyrine (APP), 3,5-dichloro-2-hydroxybenzensulfonic acid (DCHBS), xanthine, pyrogallol, putrescine dihydrochloride (PUT), d-glucose, glutamine, bovine liver catalase (9803 U/mg solid), horseradish peroxidase (HRP), butter milk xanthine oxidase, cytochrome c, bovine serum albumin, nonessential amino acids, penicillin–streptomycin, trypsin, EDTA, and 3-[4,5-dimethyl-2-thiazol-2-yl]-2,5-diphenyltetrazolium bromide (MTT) were from Sigma-Aldrich Co (St. Louis, MO, USA). Fetal bovine serum (FBS) was purchased from Wisent Inc. (St-Bruno, QC, Canada). Dulbecco’s Modified Eagle minimum essential medium (DMEM) with high glucose (25 mM) was from Gibco Life Technologies, Co (Grand Island, NY, USA). Other chemical reagents were of ACS grade and used without further purification.

### Preparation and Characterization of DAO from *Lathyrus sativus* Seedlings

Diamine oxidase was prepared as previously reported by homogenizing *Lathyrus sativus* seedlings in 50 mM phosphate buffer (pH 5.5) containing 200 mM NaCl [20]. After preparation, samples were lyophilized and stored at −20 °C until use. All the preparations were characterized for DAO activity as well as for the presence of contamination-related enzymes that may modify the level of H_2_O_2_, namely, catalase (CAT), peroxidase (POD), and superoxide dismutase (SOD). Separately, some DAO preparations were treated with sodium azide in order to inhibit traces of CAT and POD: DAO at a concentration of 19.8 mg powder per milliliter were incubated with 0.2 mg/mL sodium azide at 37 °C for 200 min in 50 mM phosphate buffer, pH 7.4.

Diamine oxidase activity was assayed at 25 °C with 3 mM putrescine in 0.1 M potassium phosphate buffer (pH 7.4) by measuring the rate of H_2_O_2_ production that forms pink adducts (ε _515 nm_ = 2.6 × 10^4^ M^−1^ cm^−1^) with 2 mM 4-aminoantipyrine (APP) in the presence of horseradish peroxidase (HRP) (10 U/mL) and of 4 mM 3,5-dichloro-2-hydroxybenzensulfonic acid (DCHBS) medium [14]. One unit (U) of DAO oxidized 1 μmol of putrescine per minute. Catalase activity was assayed by measuring the initial decrease in the absorbance of a solution of 15 mM H_2_O_2_ in 66 mM phosphate buffer, pH 7 (*ε*_240nm_ = 36 M^−1^ cm^−1^) [21]. One unit of CAT decomposed 1 μmol of H_2_O_2_ per minute. Peroxidase activity was assayed at 25 °C in 14 mM phosphate buffer, pH 6, with 40 mM pyrogallol by measuring the production rate of purpurogallin (*ε*_420 nm_ for 0.1% purpurogallin solution = 12 cm^−1^) in the presence of 8.6 mM H_2_O_2_ [22]. One unit of POD produced 1 mg of purpurogallin per minute. Protein content was estimated by the Bio-Rad protein assay reagent using bovine serum albumin as the calibration standard. Superoxide dismutase activity was assayed at 550 nm by measuring the reduction of 10 μM cytochrome c by superoxide generated during the oxidation of 50 mM xanthine by xanthine oxidase [23]. One unit of SOD decreased the rate of cytochrome c reduction by 50%.

Lyophilized DAO samples contained 0.38 ± 0.02 mg protein/mg solid, 12.25 ± 4.92 U DAO/mg solid, 1.32 ± 0.97 U CAT/mg solid, 0.93 ± 0.20 U POD/mg solid, and 13.9 ± 2.03 U SOD/mg solid.

### Measurement of Histamine Degradation and H_2_O_2_ Production

Kinetic studies of histamine degradation and H_2_O_2_ production resulting from the oxidative deamination of histamine by DAO were performed at 37 °C in Dulbecco’s modified Eagle essential minimum medium (DMEM) by incubating 2.75 or 8.5 mM histamine with 0.77, 4.62, or 9.40 mg solid/mL DAO, in the absence or presence of CAT (2941 U/mL). Histamine concentration was estimated by a modified spectrofluorimetric method (*λ*_ex_ = 360 nm; *λ*_em_ = 450 nm) [24]. Briefly, at intervals of time, 10-μL aliquots of the incubation mixture were withdrawn and added to 0.290 mL 0.1 M HCl to stop the oxidative deamination of histamine. Samples were then diluted 50 or 133 times in 2 mL 0.1 M HCl. Then, 80 μL of 5 M NaOH and 10 μL of 0.5% *w*/*v* o-phthaldialdehyde (OPT) were added. Samples were incubated at room temperature for 4 min, and the reaction was stopped with 200 μL of 3 M HCl. The concentration of histamine was estimated according to a standard curve established under the same experimental conditions.

To measure the rate of H_2_O_2_ production as a result of the oxidative deamination of histamine by DAO, 5- or 10-μL aliquots of the incubation mixture were withdrawn, added to 30 μL of a solution containing 0.8 M bromoethylamine and 25 mg/mL sodium azide and incubated at room temperature for 15 min to inhibit DAO, CAT, and POD activities. Samples were further diluted 30 times in the APP/DCHBS/HRP medium described above and incubated for 3 min at room temperature. The absorbance was then read at 515 nm, subtracted from the baseline absorbance (650 nm), and the concentration of H_2_O_2_ was estimated (*ε*_240nm_ = 36 M^−1^ cm^−1^).

### Cell Culture

The Caco-2 cell line (passages 207 to 255), obtained from Dr. A Zweibaum [16, 17], was maintained in DMEM containing 25 mM glucose and supplemented with 15% inactivated (56 °C for 30 min) fetal bovine serum (FBS), 0.1 mM nonessential amino acids, and 50 U/mL–50 μg/mL penicillin–streptomycin. Stock cultures were seeded in 75-cm^2^ culture flasks at 37 °C in a 5% CO_2_–95% humidified air atmosphere and were passaged weekly by trypsinization (0.05% trypsin–0.053 mM EDTA). For all the experiments, cells were seeded in 96-well plates (5 × 10^3^/well). The culture medium was changed every 2 days, and cells were maintained for 7 or 21 days in order to study early confluent but undifferentiated cell cultures and well-differentiated cells, respectively [18].

### Cell Viability Measurement

Cell viability was measured by the MTT colorimetric assay, which measures MTT reduction to a blue formazan product by dehydrogenases of viable cells [25]. Cells were rinsed twice with DMEM FBS-free culture medium prior to a 24-h incubation in the presence of histamine, histidine, imidazole, or mixtures of histamine (2.75 or 8.5 mM) and DAO (0.77, 4.62, or 9.40 mg solid/mL) in the presence or in the absence of 0.77 mg solid/mL CAT in DMEM FBS-free medium. At the end of the incubation period, MTT was added to each well at a final concentration of 1.2 mM (0.5 mg/mL). Cells were incubated for 2 h at 37 °C, the medium was removed, and 0.2 mL of DMSO was added to dissolve the formazan crystals. Optical density at 575 nm was measured using a Tecan SpectraFluor Plus microplate spectrophotometer (Esbe Scientific Industries Inc., Canada).

## Results

### Kinetic of H_2_O_2_ Production in FBS-Free DMEM Medium

**Fig. 1:**
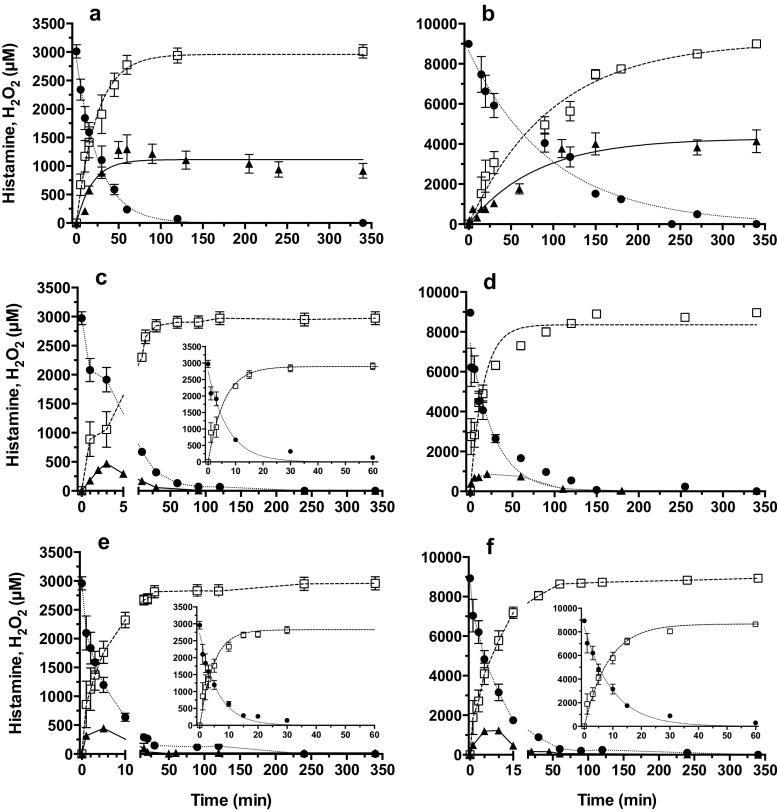
Level of histamine (*circles*) and of expected (*squares*) and measured (*triangles*) H_2_O_2_ in serum-free DMEM medium as a function of time in the presence of 0.77 mg solid per milliliter (A, B), 4.62 mg solid per milliliter (C, D), 9.40 mg solid per milliliter (E, F) DAO and 2.75 (A, C, E) or 8.5 mM (B, D, F) histamine. Inserts for C, E, F show continuing time-courses for the first 60 min. Data shown are means ± SD estimated on four different experime

Time-course of histamine consumption upon addition of DAO isolated from *Lathyrus sativus* in FBS-free DMEM well fitted a first-order decay equation (Y = Y_max_ × e^−kt^) allowing the estimation of the rate constants of consumption (k) and t_1/2_ values (ln2/k) representing time for which degradation is half-completed (Fig. 1, circles). As expected, t_1/2_ values decreased with increasing [DAO]/[histamine] ratios (Table 1). On the basis of a stoichiometry of 1:1 for histamine oxidation and H_2_O_2_ production, the expected time-course of theoretical production of H_2_O_2_ (Fig. 1, squares) was compared to measured H_2_O_2_ contents (Fig. 1, triangles). It was quite apparent that H_2_O_2_ accumulation was much lower than predicted (half to only 1/10 of the theoretical level). Moreover, in the presence of 4.62 mg/mL (Fig. 1c, d) and 9.40 mg/mL DAO (Fig. 1e, f), H_2_O_2_ generated within the first 5–20 min almost disappeared after 50–100 min. However, with DAO samples pretreated with sodium azide (that inhibits catalase but not amine oxidase activity), the measured and predicted levels of H_2_O_2_ were not significantly different (data not shown).  Thus, some contamination-related activity of CAT in DAO preparations may be responsible for the progressive degradation of generated H_2_O_2_. As far as the rate of NH_3_ accumulation is concerned, it was reversely related to the rate of histamine consumption, as expected (data not shown).

**Table 1:** Time-course of histamine degradation expressed as t_1/2_ ± SER values (min) estimated by nonlinear regression analysis according to the equation of first-order rate decay (Fig. 1): Y = Y_max_ × e^−kt^ where t_1/2_ = ln2/k and k = rate constant of histamine consumption

Histamine	Vegetal DAO		
	0.77 mg/mL	4.62 mg/mL	9.40 mg/mL
2.75 mM	18.6 ± 1.2	5.2 ± 1.0	4.3 ± 0.4
8.50 mM	68.3 ± 6.0	19.9 ± 4.5	6.8 ± 0.5

In order to completely remove H_2_O_2_ generated during histamine deamination by DAO, additional CAT was used in combination with DAO. Concentrations of CAT higher than of 200 U/mL eliminated H_2_O_2_ under our experimental conditions (data not shown). For safety and to ensure complete elimination, CAT at a concentration of 7528 U/mL was used for cell treatments.

### Effect of Vegetal DAO on Histamine Cytotoxicity

**Fig. 2:**
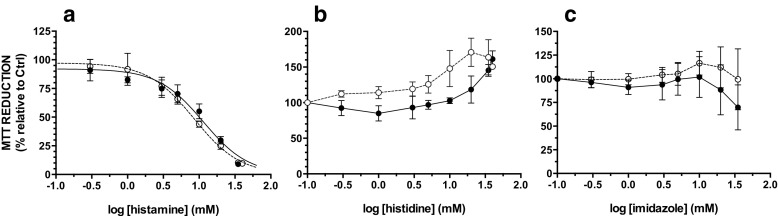
MTT activity concentration-response curve as a function of increasing concentration of histamine (**a**), histidine (**b**), and imidazole (**c**). Caco-2 cells were cultured in the presence of FBS for 7 (*open circles*) or 21 days (*filled circles*) and were then exposed to compounds for 24 h in the absence of FBS. Data shown are mean ± SD estimated on three to five independent cell cultures

When the Caco-2 cells were exposed to histamine, typical concentration-response curves of “MTT activity” were obtained as a function of increasing concentrations of histamine with LC_50_ (histamine concentration for which cell viability is half the control value) of 8.5 ± 0.9 mM on day 7 of culture and 11.8 ± 3.4 mM on day 21 of culture (Fig. 2a). Histidine did not affect cell viability up to 1 mM, and a 50% increase in “MTT activity” was measured at higher concentrations up to 30 mM (Fig. 2b). Imidazole up to 10 mM did not affect cell viability whereas a 30% decrease was obtained at 30 mM (Fig. 2c) with no significant difference between undifferentiated and differentiated cells.

**Fig. 3:**
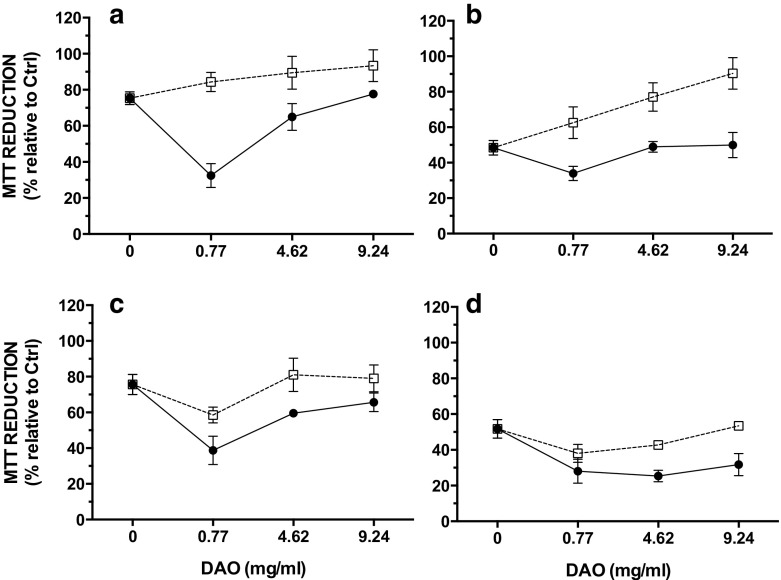
Effect of DAO and histamine on viability of cells, expressed as MTT activity. Cells were cultured in the presence of FBS for 7 (**a**, **b**) or 21 days (**c**, **d**) and were then exposed for 24 h to various concentrations of DAO and 2.75 (**a**, **c**) or 8.5 mM (**b**, **d**) histamine with (*open squares*) or without (*filled circles*) 0.77 mg solid/mL catalase in the absence of FBS. Data shown are mean ± SD estimated on six to seven independent cell cultures

The effect of the vegetal DAO on histamine toxicity was studied for 2.75 and 8.5 mM histamine which led to 25 and 50% mortality, respectively (Fig. 2a). Diamine oxidase at a concentration of 0.77 mg solid/mL significantly increased the toxicity of 2.75 or 8.5 mM histamine, lowering cell viability to about 32–38% in 7-day-old cells (Fig. 3a, c, filled circles). This effect was less evident in the presence of 4.62 mg solid per milliliter of DAO, and it did not occur at a DAO concentration of 9.24 mg solid per milliliter.

Similar results were obtained in 21-day-old cells exposed to 2.75 mM histamine, whereas in cells treated with 8.5 mM histamine, the same level of mortality was obtained, regardless of the DAO concentration (Fig. 3b, d, filled circles). The greater protection observed at higher DAO concentrations can be explained by the faster rate of histamine decomposition, which depends on DAO content and also by the faster H_2_O_2_ removal due to the presence of higher levels of contaminating CAT (i.e., 1.02, 6.10, and 12.20 U CAT for 0.77, 4.62, and 9.24 mg/mL DAO, respectively).

In all cases, the addition of exogenous CAT at a concentration of 0.77 mg solid per milliliter (7548 U/mL) efficiently protected the cells against DAO-induced histamine toxicity (Fig. 3, open squares). The protective effect of CAT was very strong in 7-day-old cells, where it efficiently decreased not only the DAO-dependent histamine toxicity but also the toxicity of histamine per se.

**Fig. 4:**
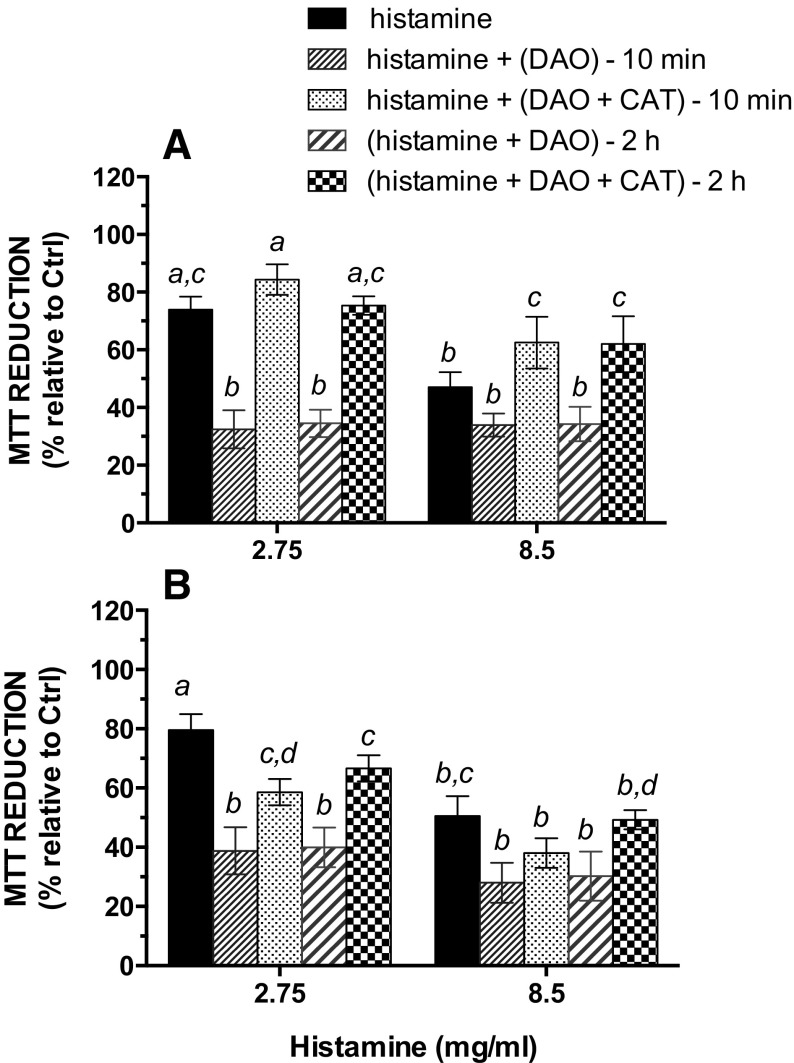
Effect of DAO and histamine on MTT activity as value of cell viability. Cells were cultured in the presence of FBS for 7 (**a**) or 21 days (**b**) and were then exposed for 24 h to 2.75 or 8.5 mM histamine, alone (*black columns*) or in the presence of 0.77 mg/mL DAO with or without 0.77 mg/mL catalase. Cells were exposed to DAO (± CAT) 10 min prior to the addition of histamine or were exposed to a mixture of DAO and histamine (with and without catalase) preincubated for 2 h at 37 °C before use for cell treatment. Data shown are mean ± SD estimated on three to six independent cell cultures. *Columns labeled with different letters* are significantly different (*p* ≤ 0.05)

To better understand the effect of combinations of DAO and CAT on histamine cytotoxicity, parallel experiments were conducted to get information on the cells’ response to the products resulting from a 24-h deamination of histamine: (1) when histamine was added to cells after a 10-min pretreatment with DAO (with or without CAT) and (2) when DAO was first preincubated with histamine (with or without CAT) in the absence of cells, followed by incubation with the cells (Fig. 4). In 7-day-old cultures, the presence of CAT afforded protection from the damage induced by 0.77 mg/mL DAO plus histamine, irrespective of the exposure protocol or the histamine concentration. However, in 21-day-old cells, the protective effect of CAT was slightly lower than in 7-day-old cells, in accordance with the moderately lower protection provided by CAT at higher levels of histamine (Fig. 3).

## Discussion

Immunomodulators and signaling and pro-inflammatory agents such as histamine are well recognized among the causative factors of intestinal inflammatory conditions and related colorectal cancer incidence. Different histamine antagonists are commercially available, but none of them effectively acts on the intestine. In contrast, vegetal DAO has been suggested as an efficient therapeutic enzyme able to metabolize histamine and evidence has been provided that it can control histamine level in different organs including the small intestine [26]. However, the catalytic activity of DAO on histamine results in the generation of toxic products such as H_2_O_2_, NH_3_, and imidazole acetaldehyde. Among these products, H_2_O_2_, a potent pro-oxidant, is particularly toxic, and for this reason we have suggested DAO in combination with CAT as therapeutic enzymes to improve the therapy of IBD and to prevent the colorectal cancer.

Histamine was found to be similarly toxic to the Caco-2 cells, independent of the differentiation status, with a LC_50_ of ≅ 10 mM (Fig. 2a). Histidine, the precursor in the biosynthesis of histamine, did not affect cell viability and even induced a 50% increase in “MTT activity” at concentrations higher than 1 and 30 mM in 7- and 21-day-old cells, respectively (Fig. 2b). MTT assay is widely used in cytotoxicity studies as well as for measuring cell proliferation. However, it was previously shown that an increase in “MTT activity” does not necessarily mean cell proliferation [27–29]. Additional experiments will be required to elucidate whether higher “MTT activity” is related to a real increase of cell proliferation or solely to an increase of cellular dehydrogenase activities. Interestingly, [^3^H]-thymidine incorporation measurements have revealed that much lower levels of histamine (1 μM) would stimulate Caco-2 cell proliferation [30], whereas colorimetric measurements similar to the MTT assay failed to demonstrate a proliferative effect [31]. Imidazole, the aromatic ring of histamine, did not significantly affect cell viability, but higher concentrations should be tested as a downward trend in MTT reduction was observed for levels higher than 30 mM, especially in differentiated cells (Fig. 2c).

Although a similar sensitivity to histamine was obtained regardless of the cell growth stage, the differentiated 21-day-old cells were more sensitive to histamine in the presence of DAO than the undifferentiated 7-day-old cells (Figs. 3 and 4). Moreover, whatever the concentration of histamine, CAT provided a higher level of protection in undifferentiated cells, whereas the protection was less important in differentiated cells. These data may suggest that by-products, other than H_2_O_2_, could be responsible for the observed differences in cell sensitivity. Thus, the responsiveness of the Caco-2 cells to imidazole acetaldehyde or ammonia, generated during the oxidative deamination of histamine, may vary with the differentiation status. However, higher levels of cellular antioxidant enzymatic activities (including CAT) have been suggested in differentiated Caco-2 cells [32]. Alternatively, differences in sensitivity to mixtures of histamine and DAO may be related to various levels of expression of endogenous DAO. Indeed, DAO does not seem to be involved in the enterocytic differentiation of the Caco-2 cells [33], but the level of DAO expression in these cells increases during the differentiation process [34]. Moreover, such as in the intestinal mucosa in vivo, significant DAO secretion is observed with differentiated Caco-2 cells [35]. Though this secretion would mainly occur through the basolateral side of the cell membrane (which is optimized in filter-grown cultures), some lateral secretion of endogenous DAO from cells grown on Petri dishes cannot be excluded. This additional extracellular DAO would lead to higher levels of H_2_O_2_ production in the culture medium of differentiated cells compared to undifferentiated cells during exposure to histamine. Moreover, and contrary to vegetal DAO, the DAO secreted from cells is not expected to be “contaminated” with CAT.

The higher toxicity of histamine obtained with 0.77 mg solid per milliliter of DAO compared to 4.62 and 9.40 mg solid/mL DAO is in agreement with the time-course data of H_2_O_2_ production (Fig. 1). Indeed, 1.1 and 4.2 mM H_2_O_2_ were still measured in the medium at the end of the 24-h incubation period with 2.75 and 8.5 mM histamine, respectively, whereas no significant levels of H_2_O_2_ could be detected in the medium after 2 h of exposure to higher DAO concentrations. Accordingly, 4.62 and 9.40 mg solid/mL DAO did not significantly increase histamine toxicity and cell viability was comparable to that measured in the presence of histamine alone, especially in undifferentiated cells (Fig. 3). Thus, deamination of histamine with low DAO produced higher and more sustained levels of H_2_O_2_ compared to high concentrations of DAO. Data obtained with the sodium azide-pretreated enzyme suggest that the rapid disappearance of H_2_O_2_ with high concentrations of DAO may be related to CAT contaminating DAO preparations, which in fact provides some protection against H_2_O_2_ toxicity.

The present data suggest that the combination of DAO with CAT has great potential to control the biological effect of histamine. Considering the turnover number of the two enzymes, appropriate concentrations are required for a fast removal of histamine without accumulation of H_2_O_2_. The presence of CAT not only promotes the clearance of toxic H_2_O_2_ but, by releasing oxygen, also favors the shift of equilibrium toward histamine decomposition in the DAO-catalyzed reaction.

## Abbreviations

DAO: Diamine oxidase (histaminase)
CAT: Catalase
SOD: Superoxide dismutase
POD: Peroxidase
APP: 4-Aminoantipyrine
DCHBS: 3,5-Dichloro-2-hydroxybenzensulfonic acid
PUT: Putrescine dihydrochloride
DMEM: Dulbecco’s modified Eagle essential medium
MTT: 3-[4,5-Dimethyl-2-thiazol-2-yl]-2,5-diphenyltetrazolium bromide
FBS: Fetal bovine serum
LC_50_: Lethal concentration leading to 50% mortality
